# Functional and internalizing disorders co-aggregate with cardiometabolic and immune-related diseases within families: a population-based cohort study

**DOI:** 10.1186/s12916-025-04293-7

**Published:** 2025-08-11

**Authors:** Olivier D. Steen, Martje Bos, Sonja L. van Ockenburg, Yiling Zhou, Ilja M. Nolte, Harold Snieder, Kenneth Kendler, Judith G. M. Rosmalen, Hanna M. van Loo

**Affiliations:** 1 Department of Psychiatry, University of Groningen, University Medical Center Groningen, Groningen, Netherlands; 2 Department of Internal Medicine, University of Groningen, University Medical Center Groningen, Groningen, Netherlands; 3 Department of Epidemiology, University of Groningen, University Medical Center Groningen, Groningen, Netherlands; 4 Virginia Institute for Psychiatric and Behavioral Genetics, Virginia Commonwealth University, Richmond, USA

**Keywords:** Fibromyalgia, Myalgic encephalomyelitis/chronic fatigue syndrome, Irritable bowel syndrome, Aetiology, Familial co-aggregation, Cardiometabolic, Atopy, Autoimmune

## Abstract

**Background:**

Functional disorders share familial risk with internalizing disorders such as generalized anxiety disorder and depression, and are comorbid with cardiometabolic and immune-related diseases. We investigated whether functional and internalizing disorders co-aggregate with these diseases in families to gain insight into the aetiology of functional and internalizing disorders.

**Methods:**

We included 166,774 subjects (aged 3–94), from the population-based Lifelines Cohort Study, a Dutch general population cohort. We defined cases for three functional disorders (myalgic encephalomyelitis/chronic fatigue syndrome; ME/CFS, fibromyalgia, and irritable bowel syndrome; IBS), two internalizing disorders (major depressive disorder; MDD and generalized anxiety disorder; GAD), cardiometabolic diseases (obesity, metabolic associated steatotic liver disease, type 2 diabetes, hypertension and cardiovascular disease) and immune-related diseases (composite measures of auto-immune disease and atopy). We used logistic regression to model the prevalence of these disorders in the general population and in participants with affected relatives. Using these prevalence estimates, we assessed familial co-aggregation with (1) recurrence risk ratios (*λ*_*R*_), and (2) familial correlations (*r*_*f*_).

**Results:**

All functional and internalizing disorders co-aggregated with immune-related diseases (*λ*_*R*_ range 1.06–1.24). ME/CFS, FM, and MDD co-aggregated with most cardiometabolic diseases (*λ*_*R*_ range 1.00–1.23). MDD, fibromyalgia, and ME/CFS showed similar familial correlation patterns with both disease groups (*r*_*f*_ range 0.12–0.44), while patterns of IBS and GAD were more variable.

**Conclusions:**

Internalizing and functional disorders share familial risk with immune-related and cardiometabolic diseases. This suggests that risk factors relevant to immune-related and cardiometabolic diseases may also be relevant for FDs. Future studies should investigate such risk factors to identify novel treatment targets.

**Supplementary Information:**

The online version contains supplementary material available at 10.1186/s12916-025-04293-7.

## Background

Functional disorders (FDs) such as fibromyalgia (FM), myalgic encephalomyelitis/chronic fatigue syndrome (ME/CFS) and irritable bowel syndrome (IBS) are characterized by somatic symptoms with poorly understood aetiology. These disorders are common, affecting up to 10% of individuals in European countries [1]. They significantly impact quality of life and functioning. Despite this, there is a lack of effective treatments, likely deriving from little insight into the mechanisms underlying the development of FDs. 

Current etiological models suggest a complex interplay of biological, psychological, and social factors contributing to the development of FDs. A systematic review identified alterations in multiple body systems, including the immune and cardiovascular systems, as relevant etiological factors [2]. Part of these factors is likely familial in origin, as twin studies have found that FDs are moderately heritable [3–5]. It is, however, unclear how this familial component specifically leads to the development of FDs and which mechanisms are involved. This is important to know, as mechanisms may be targeted with interventions.

Investigating familial co-aggregation with other classes of conditions can shed light on these mechanisms. If disorders cluster within families, this suggests that they share genetic and/or common environmental causes. Several studies found strong comorbidity, as well as familial links between FDs and internalizing disorders (IDs) [6]. IDs are characterized by inwardly directed emotional distress; common IDs include major depressive disorder (MDD) and generalized anxiety disorder (GAD) [7]. Another study found that FDs are associated with familial risk of a range of other disorders, including sleep and pain disorders and autoimmune diseases [8].

Other conditions, such as cardiometabolic diseases and atopy, are also significantly co-morbid with FDs, but no studies have explored familial coaggregation of FDs with those diseases. Significant comorbidity has been observed in the general population between metabolic syndrome and IBS [9], type 2 diabetes and FM [10], and cardiovascular disease and FM [11]. FDs are also associated with atopy [12, 13]. These disorders each have established mechanisms, such as metabolic dysfunction, inflammation, and IgE synthesis. If there are familial links between FDs and these diseases, this may imply that similar mechanisms are also involved in the development of FDs.

Here, in a large three-generational sample representative of the general population, we investigate familial co-aggregation of FDs with cardiometabolic and immune-related diseases. We choose to investigate these disease classes since they likely hold etiological relevance to FDs, and because of their availability in the dataset. Given the high rates of comorbidity between IDs and FDs, we compare these findings to familial co-aggregation between cardiometabolic and immune-related diseases with two common IDs (MDD and GAD). Finally, we estimate familial correlations of FDs and IDs with cardiometabolic and immune-related diseases.

## Methods

### Study population

We analysed data from Lifelines, which is a multi-disciplinary prospective population-based cohort study examining in a unique three-generation design the health and health-related behaviours of 167,729 persons living in the North of the Netherlands. It employs a broad range of investigative procedures in assessing the biomedical, socio-demographic, behavioural, physical, and psychological factors which contribute to the health and disease of the general population, with a special focus on multi-morbidity and complex genetics. Details on data collection and inclusion have been published elsewhere [14]. The Lifelines cohort is representative of the general population of the northern Netherlands [15].

We used data from the first wave (1A; 2007–2013) and its two follow-up questionnaires (1B and 1C, mean 2 and 3 years after inclusion, respectively), the second wave (2A; mean 4 years after inclusion), and the third wave (3A; mean 10.5 years after inclusion), its follow-up questionnaire (3B, mean 12 years after inclusion), and an add-on questionnaire on skin diseases [16] (mean 9 years after inclusion). We used data from all participants, including children, with sufficient data to ascertain case status for at least one of the studied disorders (*n* = 166,774).

For all studied disorders and diseases, we classify participants as lifetime cases or controls, meaning that they are cases if they fulfil the case definition in at least one assessment. If they do not fulfil the case definition in any non-missing assessment, they are classified as controls. Participants who are thus cases at one assessment and controls in another are considered lifetime cases and included in the analyses as such. We applied the same logic to composite phenotypic definitions; participants were not required to have complete data on all phenotypes to be a control. If they were controls at all non-missing assessments, they were coded as controls.

### Functional disorders

Data on FDs was collected during wave 2A and 3A through questionnaires assessing all symptom criteria, from which we derived diagnoses of ME/CFS, FM, and IBS. We used the 1994 Centers for Disease Control and Prevention criteria [17] for ME/CFS and the 2010 American College of Rheumatology criteria [18] for FM. We used adjusted ROME III criteria [19] for IBS to align with ROME IV (recurrent abdominal pain more than one day per week for at least 6 months, along with two additional symptoms) [20].

### Internalizing disorders

Data on current MDD (past 2 weeks) and GAD (past 6 months) were collected in waves 1A, 2A, and 3A using the Mini-International Neuropsychiatric Interview (MINI) [21], from which we ascertained diagnoses according to the DSM-IV-TR criteria [22]. Since lifetime measures of MDD and GAD were not available for many participants due to missing data, we used aggregated cross-sectional case/control status across three waves.

### Cardiometabolic phenotypes

We defined five cardiometabolic phenotypes: obesity, type II diabetes mellitus (T2D), hypertension, metabolic dysfunction-associated steatotic liver disease (MASLD) and cardiovascular disease (CVD). These disorders are common and significantly heritable [23]. Furthermore, they have a range of shared (e.g. oxidative stress, insulin resistance, low-grade inflammation) and unique mechanisms (e.g. renin–angiotensin–aldosterone system dysregulation in hypertension, beta-cell dysfunction in T2D).

#### Hypertension

Blood pressure was measured using an automatic sphygmanometer at assessment 1A, 2A, and 3A. We defined hypertension as either systolic pressure ≥ 140 mmHg, diastolic pressure ≥ 90 mmHg in any assessment, or antihypertensive use at 1A.

#### Obesity

Anthropometry was performed when participants visited the research centres at waves 1A, 2A, and 3A. We defined obesity according to the WHO definition [24], which is a BMI ≥ 30 for adults and a BMI of 2 standard deviations above the WHO reference median for participants aged below 20.

#### Metabolic associated steatotic liver disease

MASLD is a chronic disease characterized by excessive fat accumulation in the liver in the absence of secondary causes such as significant alcohol consumption. At wave 1A, γ-glutamyltransferase and triglycerides were measured in blood in a subset of participants (*N* = 58,466). Together with anthropometric data, we calculated the fatty liver index as described elsewhere [25]. We defined MASLD as a fatty liver index ≥ 60.

#### Type II diabetes mellitus

Since type 1 diabetes (T1D) and T2D have a distinct pathophysiology (autoimmune vs insulin resistance), we distinguished between these two phenotypes by combining multiple types of relevant data. Generally, we defined T2D based on self-report items, glycaemic blood abnormalities, or the use of glucose-lowering drugs, with exclusion criteria for possible T1D based on insulin use and age at onset. Not all criteria could be applied in all waves, and in those, we used adapted criteria. Detailed criteria are provided in Additional file 1: Supplementary Methods [16, 26–33].

#### Cardiovascular disease

We defined cardiovascular disease (CVD) as a composite measure of heart failure, myocardial infarction, coronary artery bypass surgery or percutaneous coronary intervention, stroke, and intermittent claudication. We used self-report items, medication, and electrocardiography data (Additional file 1: Supplementary Methods). Our definition corresponds to that used in a previous paper in Lifelines [23], but with the addition of intermittent claudication and incorporating data across all assessments.

### Immune-related diseases

We assessed two composite phenotypes of immune-related diseases, consisting of autoimmune diseases and atopy. Both involve immune dysregulation but have distinct pathophysiologies, as atopy is characterized by IgE-mediated hypersensitivity to external allergens whereas autoimmune diseases involve loss of immune tolerance causing lymphocytes to target self-antigens.

#### Autoimmune disease

Autoimmune diseases have a strong shared genetic component, but are individually relatively rare [34]. To increase power, we defined autoimmune disease as a composite measure of multiple autoimmune diseases (T1D, rheumatoid arthritis, autoimmune thyroid disease, multiple sclerosis, psoriasis, celiac disease, Crohn’s disease or ulcerative colitis). We used a combination of self-reported, laboratory, and medication data (Additional file 1: Supplementary Methods).

#### Atopy

Data on atopic diseases were collected in multiple questionnaires, with separate questionnaires for children and adults. We defined atopy as food allergy [29], asthma or eczema, based on self-report items and supported by drugs where possible (Additional file 1: Supplementary Methods).

### Pedigree data

Pedigree data in Lifelines is based on information from municipal registries, self-reported familial relationships from participants, and validated with molecular genetic data if these data were available (around *N* = 80,000 participants). Nearly two-thirds of participants (*N* = 106,282, 63.7%) had at least one first-degree relative (parent, sibling, child) in the dataset, with a median of two first-degree relatives in participants with at least one first-degree relative. A minority of participants (*N* = 33,691, 20.2%) had at least one second-degree relative (half-sibling, grandparent, grandchild, aunt, uncle, niece, nephew) in the dataset, with a median of two second-degree relatives in participants with at least one second-degree relative.

For each participant, we used pedigree data to determine if they had a first- or second-degree relative affected by the relevant disorders. We did not use data provided by the proband on their family members; thus, only individuals with a relative in the dataset with data on the studied disorders had the possibility of having an affected relative. Furthermore, individuals with relatives in the dataset may be dissimilar in other ways compared to the general population. We therefore included the number of first- and second-degree relatives in the dataset with data on the relevant studied disorder for each participant as covariates for the subsequent analyses with that disorder.

### Demographics

We included age and sex as covariates in the analyses. Sex in Lifelines is recorded from the Dutch Personal Records Database. We defined age separately for cases and controls of each phenotype. For cases, age was defined as the age at which participants first satisfied the case definition. For controls, it was defined as the last age at which participants had relevant data available. This accounts for some participants not having relevant data at some waves of data collection, for instance due to dropout or death.

### Statistics

We estimated recurrence risk ratios (*λ*_*R*_) to quantify familial co-aggregation [35]. For each pair of disorders, *λ*_*R*_ is the ratio between the prevalence of one disorder in individuals with an affected first-degree relative divided by the general population prevalence. For example, in the MDD-MASLD pair, we estimated the ratio of MDD prevalence in individuals with a first-degree relative with MASLD to MDD prevalence in the general population, and vice versa. A ratio above 1 indicates shared familial risk.

We estimated prevalences using logistic regression models, with exposure of having an affected first-degree relative and adjusted for age, age^2^ (to account for non-linear prevalence patterns by age), sex and number of first-degree relatives in the dataset. We calculate plug-in prevalence estimates as average adjusted predictions across the entire sample, and in individuals with an affected first-degree relative. We accounted for correlated measurements within families using robust standard errors with a sandwich estimator. We calculated *λ*_*R*_ as the simple ratio between prevalences, and we used the Delta method [30] to calculate the standard error of *λ*_*R*_ assuming independence between the numerator and denominator.

Next, we estimated familial correlations (*r*_*f*_), which measure the shared variance of traits attributable to familial factors, which include both genetic and common environmental effects. Since our analysis uses relatives instead of twins, our estimates capture combined genetic and common environmental causes instead of strictly genetic causes [36].

We estimated familial correlations using the Wray & Gottesman method [37], which is based on the liability threshold model, where disease status is determined by an underlying normally distributed liability [38]. The estimation depends on prevalence estimates in the general population and in individuals with affected first-degree relatives, as well as with affected second-degree relatives. We adjusted both models for the number of first- or second-degree relatives in the dataset with data on the phenotype. Details and equations are provided in Additional file 1: Supplementary Methods, while an example R script for estimating marginalized prevalences, recurrence risk ratios, and familial correlations is also provided (Additional file 2).

### Preregistration, inference criteria and reporting

We preregistered our analysis plan (https://osf.io/kj7dx) with subsequent modifications to phenotype definitions and multiple testing corrections. Changes are detailed in Additional file 1: Supplementary Methods.

We tested directional hypotheses for both recurrence risk ratios (H0: *λ*_*R*_ = 1, H1: *λ*_*R*_ > 1) and for familial correlations (H0: *r*_*f*_ = 0, H1: *r*_*f*_ > 0). Because we do not expect any effect in the other direction, we evaluated one-sided tests. These amounted to a total of 105 tests. Since these tests are not independent, and our aim is exploratory (to identify novel etiological associations) controlling the family-wise error rate would be too conservative. We controlled the false discovery rate at 0.05 across all tests using the Benjamini–Hochberg procedure [39]. We reported estimates with only the lower bounds of one-sided 95% confidence intervals (CIs), as the upper bound is infinite for *λ*_*R*_*s* and 1 for r_f_s and not informative. This is the case because the CI in this case is defined by only a lower critical region, and the sampling distribution's probability density extends infinitely in the positive direction (but correlations have a theoretical maximum value of 1) [30].

### Sensitivity analysis

We conducted a sensitivity analysis to assess if our results were influenced by misclassification of FDs. We excluded participants that also reported having conditions that may present with similar symptoms or are mentioned as exclusionary in diagnostic guidelines [17]. These were multiple sclerosis, dementia, schizophrenia, or an eating disorder for ME/CFS; ulcerative colitis, Crohn’s disease, or coeliac disease for IBS; rheumatoid arthritis for FM; and hepatitis, cancer, or heart failure for all FDs.

## Results

We included data of 166,774 participants that had data available on at least one disorder (Table 1). More than half (57.9%) of participants were female. Mean age at first included assessment was 42.5 years (standard deviation 15.7), with a wide age range (3–93). Over half of participants (63.7%) had a first-degree relative in the dataset, while 20.2% had a second-degree relative in the dataset.

**Table 1:** Characteristics of study population

***N***	166,774
**Mean age (SD)**	42.42 (15.59)
**Age range**	3–94
**Male sex (%)**	70,151 (42.1%)
**First-degree relative in dataset (%)**	106,282 (63.7%)
**Second-degree relative in dataset (%)**	33,691 (20.2%)
Median first-degree relatives in dataset* (IQR)	2 (1–3)
Median second-degree relatives in dataset* (IQR)	2 (1–3)
**Hypertension**	62,033 (38.1%)
**CVD**	7862 (5.1%)
**T2D**	7966 (5.4%)
**Obesity**	34,525 (20.9%)
**MASLD**	13,167 (22.5%)
**Autoimmune disease**	15,367 (9.9%)
**Atopy**	26,569 (16.3%)
**MDD**	7266 (4.8%)
**GAD**	13,868 (9.1%)
**FM**	7982 (8.2%)
**ME/CFS**	4866 (4.7%)
**IBS**	7703 (7.5%)
**FM after exclusion**	6340 (6.6%)
**ME/CFS after exclusion**	3977 (3.9%)
**IBS after exclusion**	6427 (6.3%)

*In participants who had at least one relative in the dataset. Abbreviations: *SD* standard deviation, *IQR* interquartile range, *MASLD* metabolic associated steatotic liver disease, *T2D* type 2 diabetes, *CVD* cardiovascular disease, *ME/CFS* myalgic encephalomyelitis/chronic fatigue syndrome, *FM* fibromyalgia, *IBS* irritable bowel syndrome, *MDD* major depressive disorder, *GAD* generalized anxiety disorder

### Recurrence risk ratios

**Fig. 1:**
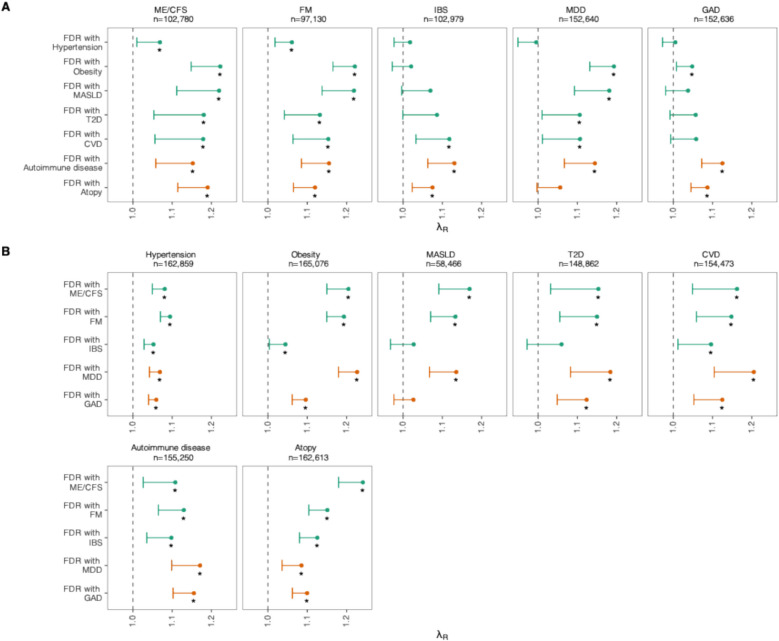
Displayed are recurrence risk ratios (*λ*_*R*_) and the lower bound of one-sided 95% confidence intervals for the ratio **A** between prevalences of IDs/FDs (name and sample size above plot) in the general population and in individuals with a first-degree relative affected by cardiometabolic (green) or immune-related diseases (orange), and **B** between prevalences of cardiometabolic/immune-related diseases in the general population and in individuals with a first-degree relative affected by an FD (green) or ID (orange). Stars denote statistical significance at false discovery rate-adjusted *p*-value < 0.05. Abbreviations: FDR, first-degree relative; MASLD, metabolic associated steatotic liver disease; T2D, type 2 diabetes; CVD, cardiovascular disease; ME/CFS, myalgic encephalomyelitis/chronic fatigue syndrome; FM, fibromyalgia; IBS, irritable bowel syndrome; MDD, major depressive disorder; GAD, generalized anxiety disorder

We estimated recurrence risk ratios to examine patterns of familial co-aggregation between IDs/FDs and cardiometabolic and immune-related diseases (Fig. 1, Additional file 3: Table S2).

We found significant familial co-aggregation between IDs/FDs and cardiometabolic diseases, but with differences between specific IDs and FDs. Estimates of familial co-aggregation with cardiometabolic disorders were broadly similar for MDD, FM, and ME/CFS. Having a first-degree relative with obesity or MASLD was associated with increased prevalence of MDD, FM, and ME/CFS, and vice versa (*λ*_*R*_ range 1.13–1.23). Patterns were similar for co-aggregation between MDD, ME/CFS, and FM with CVD and T2D (*λ*_*R*_ range 1.11–1.21). Hypertension co-aggregated in families with all IDs/FDs (*λ*_*R*_ range 1.01–1.10), except for hypertension in a first-degree relative and MDD (*λ*_*R*_ 1.00, lower 95% CI 0.95).

We also found significant familial co-aggregation between IDs/FDs and immune-related diseases. Atopy co-aggregated strongest with FDs and GAD (*λ*_*R*_ range 1.08–1.24). For MDD and atopy, co-aggregation was small, while the recurrence risk of MDD for atopy in a first-degree relative was not significant (*λ*_*R*_ = 1.06, lower 95% CI 1.00). Autoimmune disease was associated with increased prevalence of all IDs and FDs (*λ*_*R*_ range 1.13–1.16), while having a first-degree relative with an ID or FD was associated with increased prevalence of autoimmune disease (*λ*_*R*_ range 1.10–1.17).

For IBS and GAD, we observed less familial co-aggregation with cardiometabolic diseases. Only CVD in a first-degree relative was associated with increased prevalence of IBS (*λ*_*R*_ 1.12; lower 95% CI 1.03), while only obesity in a first-degree relative was associated with the prevalence of GAD (*λ*_*R*_ 1.05; lower 95% CI 1.01). However, having a first-degree relative with IBS was associated with increased prevalence of hypertension, CVD, and obesity (*λ*_*R*_*s* range 1.04–1.10), but not T2D or MASLD. Having GAD in a first-degree relative was associated with increased prevalence of all cardiometabolic disorders except for MASLD (*λ*_*R*_ range 1.06–1.13).

### Familial correlations

**Fig. 2:**
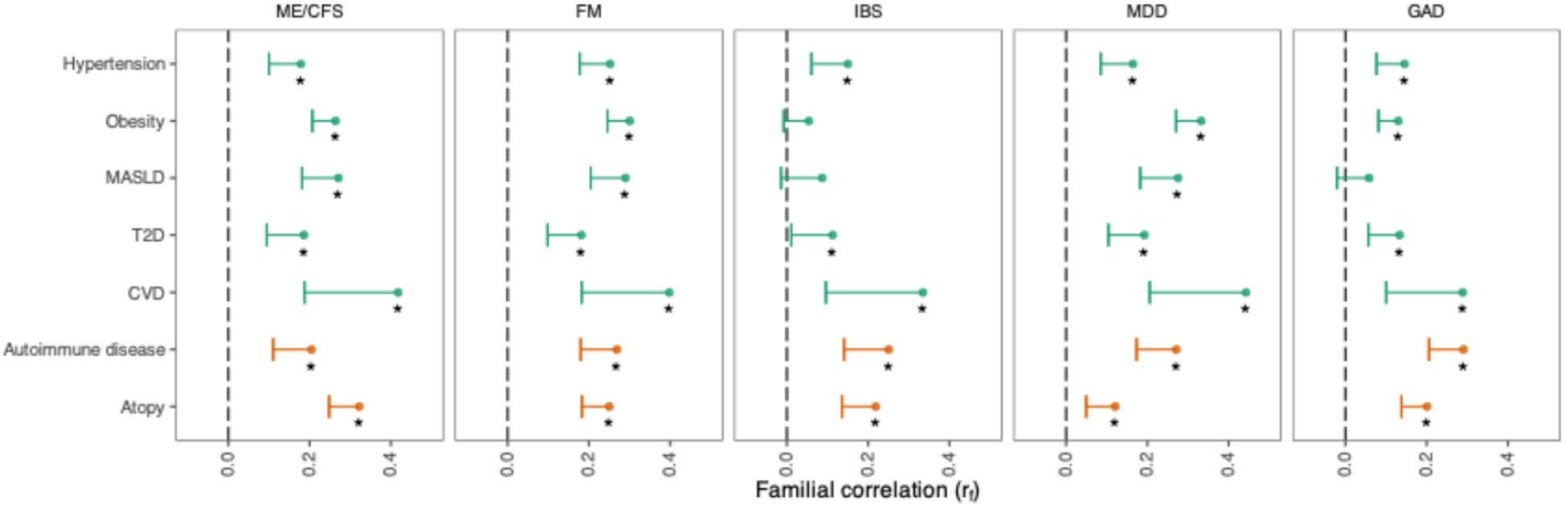
Displayed are familial correlations (*r*_*f*_) with the lower bound of one-sided 95% confidence intervals between IDs/FDs (above plot) and cardiometabolic (green) and immune-related diseases (orange). Stars denote statistical significance at false discovery rate-adjusted *p*-value < 0.05. Abbreviations: MASLD, metabolic associated steatotic liver disease; T2D, type 2 diabetes; CVD, cardiovascular disease; ME/CFS, myalgic encephalomyelitis/chronic fatigue syndrome; FM, fibromyalgia; IBS, irritable bowel syndrome; MDD, major depressive disorder; GAD, generalized anxiety disorder

We found significant positive familial correlations between most IDs/FDs and cardiometabolic and immune-related disorders (Fig. 2, Additional file 3: Table S3). ME/CFS, FM, and MDD had significant small to moderate familial correlations with all cardiometabolic and immune-related disorders (*r*_*f*_ range 0.16–0.44). All IDs and FDs had small to moderate familial correlations with both atopy and autoimmune disease (*r*_*f*_ range 0.12–0.32). For IBS, we found significant familial correlations with hypertension, T2D, and CVD (*r*_*f*_ range 0.11–0.34), but not with obesity or MASLD. For GAD, we found significant familial correlations with all cardiometabolic disorders except for MASLD (*r*_*f*_ range 0.13–0.29).

### Sensitivity analysis

We conducted a sensitivity analysis to account for potential misclassification (Additional file 1: Figs. S1–2. Fig. S1—Recurrence risk ratios. Fig. S2—Familial correlations; Additional file 3: Tables S4–5). Results were similar; however, with more uncertainty for some recurrence risk ratios. The confidence interval for having a first-degree relative with ME/CFS or IBS and the prevalence of autoimmune disease was wider and no longer significant, while having ME/CFS in a first-degree relative was no longer significantly associated with increased prevalence of T2D. However, having a first-degree relative with autoimmune disease or with T2D was robustly associated with increased prevalence of all FDs. Familial correlation patterns were similar.

## Discussion

In this large population-based study, we found robust familial co-aggregation between FDs/IDs and two major disease groups. First, we found significant familial co-aggregation between all studied FDs/IDs and immune-related diseases. This is supported by increased prevalence of IDs and FDs in individuals with a first-degree relative with autoimmune disease or atopy, an increased prevalence of autoimmune disease and atopy in those with a first-degree relative with an ID or FD, and significant familial correlations between IDs/FDs and both atopy and autoimmune disease. Second, we also found significant familial co-aggregation of IDs/FDs and cardiometabolic diseases, with the most robust co-aggregation with ME/CFS, FM and MDD. This is indicated by an increased prevalence of MDD, FM and ME/CFS in those with a first-degree relative with obesity, MASLD, T2D and CVD, an increased prevalence of cardiometabolic diseases in individuals with a first-degree relative with MDD, FM and ME/CFS, and significant familial correlations between MDD, ME/CFS and FM with all studied cardiometabolic diseases.

### Comparison with previous studies

For MDD and GAD, which have received considerably more research interest than FDs, our results agree with previous studies. Register-based studies found familial co-aggregation between MDD/GAD and autoimmune diseases [40, 41] and atopy [42], and between MDD and cardiometabolic diseases [43]. Also, molecular genetic data suggests that shared genetics explains part of this shared familial risk. Significant genetic correlations have been observed between anxiety and several immune-related diseases [44], MDD and hypothyroidism [45], and MDD and several cardiometabolic diseases [46]. Furthermore, both genetic correlation and familial co-aggregation have been found between stress-related disorders (which are often comorbid with IDs and FDs) and autoimmune diseases [47].

For FDs, much less comparable studies have been performed. One prior study investigated familial genetic risk scores of autoimmune diseases in FDs [8]. It found higher estimates of familial genetic risk of autoimmune diseases in FM, IBS, and ME/CFS compared to MDD. This is somewhat different from our findings, where the degree of familial co-aggregation with autoimmune diseases was similar across all FDs and IDs, but we did not test for differences between FDs and IDs. The different findings could also derive from a higher severity of FM in its sample, since its prevalence of FM based on registry data was much lower (0.4%) compared to that in our study (8.2%).

In a genome-wide association study for IBS, no significant genetic correlations were found between IBS and immune-related diseases, while we found significant familial co-aggregation and familial correlations between IBS and immune-related diseases [48]. This study, however, excluded patients with gastrointestinal autoimmune diseases from the IBS disease definition, which could attenuate an autoimmune genetic signal. Another explanation for the discrepancy could be that, for IBS, the observed familial association we found primarily derives from shared common environmental causes. However, another genome-wide association study for IBS [49] found significant genetic correlations of IBS with cardiovascular diseases, which suggests that the shared familial risk that we identified partly derives from genetic factors.

We are unable to directly compare prevalence rates between our study and other studies. This is because we used heterogeneous aggregated prevalences to maximize case finding. Although prevalence rates are important in many epidemiological studies, the prevalences are less important for the research questions we aim to answer. This is because our target of inference is a ratio of prevalences (*and both numerator and denominator are similarly aggregated*), and not the prevalences themselves.

### Interpretation and implications

Our findings suggest that IDs and FDs may share genetic and/or common environmental risk factors with immune-related and cardiometabolic diseases. These risk factors could, for example, include shared genes, other biological factors, environmental exposures, or lifestyle factors. For MDD, there is some mechanistic evidence for the involvement of such risk factors, and they are relevant to the understanding and treatment of MDD. These include biological factors such as leptin/insulin dysregulation [50], oxidative stress [51] and gut dysbiosis [52], but also lifestyle factors such as diet [53] and physical activity [54]. Our findings suggest that similar risk factors could be identified for FDs.

It is possible that such risk factors are more important in subgroups of individuals with FDs, compared to FDs as a whole. The familial effects we identified at the group level were small to moderate. Furthermore, there is heterogeneity in FDs [55, 56]. Possibly, subtypes of FDs could be identified, in which there is an important role of risk factors shared with cardiometabolic and immune-related diseases. Similarly, for MDD there is evidence for an immunometabolic subtype [46]. Future studies may be able to identify such subgroups, for instance by investigating FD patients with a high genetic risk of cardiometabolic or immune-related diseases through molecular genetic data, or through data on relatives. Furthermore, data such as comorbid diseases, or exposure to an infectious trigger, may be used to identify homogeneous subgroups, for instance using factor mixture modelling [57].

A similar point applies to the aggregate autoimmune diseases phenotype. We included all autoimmune diseases available in the dataset to maximize power, which makes it unclear if specific autoimmune diseases have a stronger association than others. Some autoimmune diseases are more prevalent than others; they may have a stronger impact on the observed familial co-aggregation. However, less common autoimmune diseases can also still make relatively large contributions in case of high heritability. Future studies may be able to identify specific roles of immune dysregulation in the aetiology of IDs/FDs.

Our study does not provide insight into specific genetic or environmental causal pathways underlying the observed familial associations. For instance, cardiovascular disease in a parent may cause MDD in offspring through shared genetic risk but also through stressful life experiences, while a shared unhealthy lifestyle may contribute to major depression in the parent and cardiovascular disease in the offspring, or FDs may prompt sedentary behaviour leading to obesity in offspring. Future studies using methods such as twin-sibling modelling, molecular genetic studies, or bidirectional causal modelling [58], and studies taking into account the temporal order of disease onsets may give insight into causal mechanisms.

Our findings have implications for research and clinical practice. Our finding of shared familial risk factors between IDs/FDs and cardiometabolic and immune-related diseases suggests new directions for research into the aetiology of FDs, for instance, in investigating shared genetic risk factors, but also shared family environmental risk factors. A further understanding of these familial risk factors will be helpful for clinicians in explaining an FD diagnosis to patients.

Second, we found similarities but also differences in patterns of familial co-aggregation between FDs and IDs. Previous studies have suggested that multiple individual FD diagnoses can be captured under a single umbrella diagnosis, such as functional somatic syndrome [59] or bodily distress syndrome [60]. Our study both supports and opposes this approach, as we found that the three types of FDs have similar familial associations with immune-related diseases but dissimilar associations with cardiometabolic diseases.

Third, our findings suggest that a broad family history inquiring into IDs, FDs, cardiometabolic and immune-related diseases may be a relevant part of the clinical approach to FDs and IDs. This could, for instance, be relevant for understanding etiological contributions in an individual patient, but also for identifying individuals at risk of cardiometabolic and immune-related diseases in the ID/FD patient population.

### Strengths and limitations

Strengths of our study include the use of a large population-based cohort and the use of diagnostic criteria for defining FDs and IDs. This reduces the impact of diagnostic biases and help-seeking behaviour compared to hospital and registry data and self-reported diagnoses of FDs. Furthermore, the family-based design prevents the need to rely on unreliable information from probands about their relatives.

We note however also some limitations. First, some of the cardiometabolic and immune-related phenotypes depend on aggregated data with variable quality and missingness rates. This could cause that some cases for these diseases might be classified as controls, if they only participated in assessments in which they did not fulfil case definitions. This would cause an underestimation of prevalences in both the general population and in individuals with affected relatives; therefore not biasing estimates in any direction, but it would decrease power. There is furthermore likely some heterogeneity due to the aggregation, meaning that the familial effects we found are driven by some of the aggregated diseases.

Another source of potential bias is help-seeking bias, which is relevant for measures that depend on self-reported doctor diagnoses. Since individuals with FDs may be more likely to present to a clinician and receive diagnostic interventions, these individuals will be more likely to receive additional diagnoses—leading to comorbidity and familial co-aggregation. We minimized the impact of this bias by ascertaining FDs based on symptom questionnaires and not diagnoses, and by ascertaining most cardiometabolic and immune-related diseases through measures that are not self-reported doctor diagnoses.

Furthermore, many participants did not have any relatives in the dataset. These are assumed to not have an affected relative and therefore counted only as part of the general population. However, if they do have a relative affected by an ID/FD or cardiometabolic/immune-related disease (and for some subjects this is likely since these are common disorders), any increase in risk that they have for the disease due to their affected relative will only increase the estimated prevalence in the general population, leading to an underestimation of *λ*_*R*_. We addressed this limitation by including the number of relatives in the analyses as a covariate.

Last, our uncertainty estimates of *λ*_*R*_ are likely too conservative. This is due to our calculation of the standard error of *λ*_*R*_, which assumes that the prevalence in the general population and in individuals with an affected relative are independent. However, this is not the case, since individuals with affected relatives are also part of the general population. This does not bias the estimated *λ*_*R*_, but it increases the width of confidence intervals and thus increases the chance of a false negative finding. We tested the impact of this limitation by calculating a bootstrap confidence interval for one *λ*_*R*_, which only provided marginally smaller confidence intervals; but this is a computationally expensive procedure. Therefore, we decided to only report confidence intervals calculated using the Delta method. 

## Conclusions

Our study reveals shared familial risk between FDs/IDs and major disease groups. We found that MDD, ME/CFS, and FM robustly share familial risk with cardiometabolic diseases, while all studied IDs and FDs have familial links with immune-related diseases. Our findings suggest that the aetiology of FDs may include risk factors shared with common somatic diseases, which could be identified in future studies.

## Supplementary Information

Additional file 1: Supplementary Methods and Figures. This file contains supplementary methods and figures as referenced in the text.

Additional file 2. This file contains an example R script for estimating marginalized prevalences, recurrence risk ratios and familial correlations. This is identical to the script we used, but for a single phenotype pair to prevent redundancy.

Additional file 3. This file contains supplementary tables as referenced in the text.

## Data Availability

Data may be obtained from a third party, Lifelines (14), and are not publicly available. Researchers can apply to use the Lifelines data used in this study. More information about how to request Lifelines data and the conditions of use can be found on their website (https://www.lifelines.nl/researcher/how-to-apply). An example of the R code used in this study is included in Additional file 2.

## Abbreviations

ID: Internalizing disorder
FD: Functional disorder
MASLD: Metabolic associated steatotic liver disease
T2D: Type 2 diabetes
CVD: Cardiovascular disease
ME/CFS: Myalgic encephalomyelitis/chronic fatigue syndrome
FM: Fibromyalgia
IBS: Irritable bowel syndrome
MDD: Major depressive disorder
GAD: Generalized anxiety disorder
