# Supplementary material for: Functional and internalizing disorders co-aggregate with cardiometabolic and immune-related diseases within families: a population-based cohort study
Source: BMC Med. 2025 Aug 11;23:469. doi: 10.1186/s12916-025-04293-7 (PMC12337435; doi:10.1186/s12916-025-04293-7)
Supplement: Supplementary file 1 — Additional file 1: Supplementary Methods and Figures. This file contains supplementary methods and figures as referenced in the text. [file 12916_2025_4293_MOESM1_ESM.docx]

Supplementary material for manuscript:

**Functional and Internalizing Disorders Co-Aggregate with Cardiometabolic and Immune-Related Diseases Within Families: A Population-Based Cohort Study**

Additional File 1

Contents:

Supplementary Methods

Supplementary Figures

Supplementary Methods

*Internalizing disorders*

Data collection in Lifelines started in 2006 before the publication of the DSM-5 (2013) and therefore the DSM-IV-TR criteria were used. The MINI was administered as an interview by a trained nurse in the first wave of data collection. The second wave used a digital questionnaire on-site, while the third wave used a digital questionnaire completed at home.

*Medication*

Lifelines participants were asked to report on their medication in the 1A questionnaire. Participants were also asked to bring their actual drugs, which were registered by research staff. Next, medication data was coded using the Anatomical Therapeutic Chemical (ATC) Classification System using ATC-coding software. See Additional File 2: Supplementary Table 1 for an overview of ATC codes.

*Type 2 diabetes*

We defined T2D according to any of the following criteria:

1) use of glucose-lowering drugs based on ATC codes at 1A

2) self-reported T2D with also self-reported use of glucose-lowering drugs at 1A

3) self-reported diabetes and age>30 at 1B-C (these questionnaires did ask type of diabetes).

3) elevated glycaemic laboratory values (fasting glucose >7mmol/L or HbA1c >6.5%) at 1A, 2A and 3A

4) self-reported T2D at 2A, 3A-B.

To prevent misclassification of other types of diabetes, we coded participants who had T2D according to the above definition, but any of the below criteria, as missing:

1) participants who had age <30 and either did not self-report diabetes or had missing data on self-reported diabetes. (1A, 2A and 3A)

2) participants who reported another type of diabetes (1A, 2A, 3A)

3) participants who had diabetes onset age<30 or current age<30 and treated their diabetes only with insulin (based on ATC codes or self-report) (at 1A)

4) pregnant participants (at 1A)

*Cardiovascular diseases*

We classified participants as cases if any of these criteria were met:

1) myocardial infarction at 1A and 2A based on ECG signs, according to a previous definition (26),

2) heart failure at 1A based on self-reported heart failure and supporting treatment (either self-reported pacemaker, drugs or transplantation or based on ATC codes).

3) self-reported coronary artery bypass graft surgery or percutaneous coronary intervention at 1A, 3A and 3B,

4) self-reported stroke at 1A-C, 2A, 3A-B or

5) self-reported intermittent claudication at 1B-C, 2A, 3A-B.

*Autoimmune diseases*

We defined case status for autoimmune diseases based on any of the following criteria:

1) self-reported T1D at 1A, 2A, 3A-B,

2) rheumatoid arthritis based on the combination of self-reported rheumatoid arthritis with supporting data (27) (use of a non-steroidal anti-inflammatory drug, disease-modifying antirheumatic drug or glucocorticoid; and having visited a medical specialist in the last year) at 1A, or

3) rheumatoid arthritis based on anti-cyclic citrullinated peptides > 5 U/mL at 1A,

4) self-reported or confirmed use of thyroid medications at 1A,

4) biochemical hypothyroidism (thyroid-stimulating hormone > 4 mU/L, free thyroxine < 11.0 pMol/L) or hyperthyroidism (thyroid-stimulating hormone < 0.5 mU/L, free thyroxine > 19.5 pMol/L) at 1A,

5) self-reported multiple sclerosis at 1A,

6) self-reported psoriasis at 1A and 3A,

7) self-reported celiac disease, Crohn’s disease, or ulcerative colitis at 1A-C, 2A, 3A-B.

*Atopy*

We defined atopy as any one of the following criteria were met:

1) self-reported physician diagnosed asthma in adults at 1A, or

2) reporting two asthma symptoms (out of three; wheeze, dyspnoea at rest, nocturnal dyspnoea) supported by use of asthma medication based on ATC codes in adults at 1A or supported by self-reported use of inhaled corticosteroids in adults at 2A.

3) asthma in children at 2A and 3A based on physician diagnosed asthma reported by parent or caregiver.

4) eczema in children at 2A and 3A based on physician diagnosed eczema reported by parent or caregiver.

5) eczema in adults in an add-on questionnaire based on either physician diagnosed asthma, or based on an adaptation of UK Working Party criteria (16,28) (having an itchy rash in lifetime with supporting data (three or more of the following conditions: a history of general dry skin in the past year, an onset under the age of two, a history of skin creases involvement, a history of asthma or hay fever),

6) food allergy according to a published definition in all age groups (29).

*Demographics*

In the case of cardiometabolic and immune-related diseases, we combined data from separate measurements and questionnaires within one wave that could be completed up to several months apart. Here we used the age at the last included visit as the age for that wave. For IDs and FDs this was not necessary as their definitions did not require combining data across measurements.

*Calculation of the recurrence risk ratio and its standard error*

We calculated the recurrence risk ratio λ_R_ as a measure of familial co-aggregation. Here it is the ratio between the marginalized prevalence in individuals with an FDR by one disorder (K_R_), and the prevalence of another disorder in the general population (K):

Equation 1:

$$\lambda_{R}= \frac{K_{R}}{K}$$

We used the Delta method to approximate the standard error of λ_R_. According to the Delta method (30), the standard error of a differentiable function g(K, Kr) where K and Kr are random variables, can be approximated as shown below.

Equation 2:

$$var\left( g\left( K,K_{r} \right) \right)\approx\left( \frac{\partial g}{\partial K_{r}} \right)^{2}*var\left( K_{r} \right)+ \left( \frac{\partial g}{\partial K} \right)^{2}*var\left( K \right)+2{\left( \frac{\partial g}{\partial K_{r}} \right)\left( \frac{\partial g}{\partial K} \right)*cov(K, K_{r})}$$

The partial derivatives of g(Kr, K) = Kr/K are shown below.

Equation 3:

$$\frac{\partial g}{\partial K_{r}}=\frac{1}{K}$$

Equation 4:

$$\frac{\partial g}{\partial K}=\frac{-K_{r}}{K^{2}}$$

We assumed independence between prevalences, meaning the covariance term is dropped. After also substituting the partial derivatives, the variance can be approximated by the equation below.

Equation 5:

$$var\left( g\left( K, K_{r} \right) \right)\approx\left( \frac{1}{K} \right)^{2}*var\left( K_{r} \right)+\left( -\frac{K_{r}}{K^{2}} \right)^{2}*var(K)$$

To get the standard error, we substitute variances for squared standard errors, take the square root and simplify, as shown blow.

Equation 6:

$$se(g\left( K,Kr \right))\approx\sqrt{\frac{{\mathrm{se}\left( \mathrm{Kr} \right)}^{2}}{K^{2}}+ \frac{\mathrm{Kr}^{2}*{\mathrm{se}\left( K \right)}^{2}}{K^{4}}}$$

*Software*

We performed all analyses in R (31). We used the *avg_predictions* function from the *marginaleffects* R package to estimate prevalences (32), as well as the *vcovCL* function from the *sandwich* R package to compute robust standard errors (33). An example script for estimating prevalences and calculating derived quantities is provided in Additional File 3.

*Preregistration and inference criteria*

We made several changes to our analysis compared to our preregistration (osf.io/kj7dx), we provide here an overview and explanation of these changes.

1. Type II diabetes definition

Initially we defined type 2 diabetes as self-reported T2D with supporting data (use of self-reported glucose-lowering medication, fasting plasma glucose level ≥ 7.0 mmol/L, and/or HbA1c ≥ 6.5%), excluding a group of potential type 1 diabetes cases (under 30 years of age or have an age at onset <30 years and use insulin). We were able to use this definition at 1A, however other waves had more limited data, not including medication data and some not asking about the type of diabetes. We adjusted our definition to make the best possible use of all the available data. Furthermore, we realized that also type II diabetics can use insulin, and this is also possible at relatively young ages. Therefore, we adjusted our definition to using *only* insulin and onset before 30 to exclude potential type 1 diabetes cases at 1A.

1. Cardiovascular disease definition

We changed this definition by not including the use of thrombocyte aggregation inhibitors as a criterion, to align our definition with other studies using the same dataset, and as thrombocyte aggregation inhibitors are likely not specific enough for cardiovascular diseases. We also include self-reported pacemaker, heart transplant or drug use for heart failure as supporting data for heart failure, since we became aware that Lifelines has collected this data.

1. Sensitivity analysis

Our original analysis plan stated we would account for misclassification through a sensitivity analysis that would subset to individuals who were controls for disorders of the affected relatives. However, this approach was flawed, as it would eliminate the covariance between disorders—however covariance would be expected if disorders share familial causes. Additionally, this method would reduce generalizability by using a selected population. We therefore modified our sensitivity analysis to align with another approach used in our lab, excluding only disorders that could be misclassified based having similar symptoms from the FD case definition.

1. Multiple testing

Our preregistration stated we will not correct for multiple testing as this is an exploratory study. Since we considered that this not rigorous enough, we decided to correct for multiple testing using the false discovery rate. This only makes our inference criteria more stringent, so we did not consider this incompatible with open scientific practices. We reported unadjusted p-values in Additional File 2: Supplementary Tables.

1. Imputation

In the preregistration we stated we aimed to use multiple imputation with chained equations, in case a participant has data on at least one measurement. However, since some measurements were only available in a subset of participants, this would mean a substantial amount of imputation of binary phenotypes. Since we had sufficient power using only the non-missing data, we decided against imputation.

Supplementary Figures

***Supplementary Figure 1:*** Displayed are recurrence risk ratios (λ_R_) and one-sided 95% confidence intervals for the ratio **A:** between prevalences of FDs (name above plot) in the general population and in individuals with a first-degree relative affected by cardiometabolic and immune-related diseases (y-axis), and **B:** between prevalences of cardiometabolic/immune-related diseases (name above plot) in the general population and in individuals with a first-degree relatives affected by FDs (y-axis). We estimated recurrence risk ratios in samples excluding individuals that had a somatic disorder that could potentially be misclassified as an FD. FDR: first-degree relative, MASLD: metabolic associated steatotic liver disease, T2D: type 2 diabetes, CVD: cardiovascular disease, ME/CFS: myalgic encephalomyelitis/chronic fatigue syndrome, FM: fibromyalgia, IBS: irritable bowel syndrome, MDD: major depressive disorder, GAD: generalized anxiety disorder.

**

**Supplementary Figure 2***:* Displayed are familial correlations (r_f_) with one-sided 95% CI between FDs (above plot) and cardiometabolic (green) and immune-related diseases (orange). We estimated familial correlations in samples excluding individuals that had a somatic disorder that could potentially be misclassified as an FD. MASLD: metabolic associated steatotic liver disease, T2D: type 2 diabetes, CVD: cardiovascular disease, ME/CFS: myalgic encephalomyelitis/chronic fatigue syndrome, FM: fibromyalgia, IBS: irritable bowel syndrome.
